# Draft Genome Sequences of *Tersicoccus phoenicis* DSM 30849^T^, Isolated from a Cleanroom for Spacecraft Assembly, and *Tersicoccus* sp. Strain Bi-70, Isolated from a Freshwater Lake

**DOI:** 10.1128/genomeA.00079-17

**Published:** 2017-03-30

**Authors:** Yu Nakajima, Susumu Yoshizawa, Keiji Nakamura, Yoshitoshi Ogura, Tetsuya Hayashi, Kazuhiro Kogure

**Affiliations:** a Laboratory of Marine Microbiology, Department of Marine Ecosystem Dynamics, Division of Marine Life Science, Atmosphere and Ocean Research Institute, The University of Tokyo, Kashiwa, Chiba, Japan; b Department of Bacteriology, Faculty of Medical Sciences, Kyushu University, Fukuoka-shi, Fukuoka, Japan

## Abstract

Here, we report the draft genome sequences of *Tersicoccus phoenicis* DSM 30849^T^, isolated from a spacecraft assembly cleanroom at the National Aeronautics and Space Administration (NASA), and *Tersicoccus* sp. strain Bi-70, isolated from Lake Biwa, the largest lake in Japan. These genome sequences facilitate our understanding of the adaptation of these closely related strains to different habitats.

## GENOME ANNOUNCEMENT

*Tersicoccus phoenicis* DSM 30849^T^ is an aerobic Gram-positive bacterium belonging to the phylum *Actinobacteria*. It was first isolated from a National Aeronautics and Space Administration (NASA) spacecraft cleanroom which was thought to be biologically contaminant free (1). On the other hand, *Tersicoccus* sp. strain Bi-70 NBRC 112423 was isolated from Lake Biwa, the largest lake in Japan. Currently, the genus *Tersicoccus* includes two described species, *T. phoenicis* (type strain, DSM 30849^T^) and *Tersicoccus solisilvae* (KCTC 33776^T^), which was isolated from forest soil (2).

The genomic DNA of both strains was extracted using phenol-chloroform and ethanol precipitation (3). The Kapa HyperPlus kit (Kapa Biosystems) was used for library preparation, and paired-end sequences (300 bp of each end) were obtained on the MiSeq instrument with the MiSeq reagent kit version 3 (Illumina). Genome assembly was performed using *Platanus* version 1.2.4 (4). The assembled sequences were annotated using the NCBI Prokaryotic Genome Annotation Pipeline (PGAP) (5) and reviewed with the RAST version 2.0 (http://rast.nmpdr.org). The 16S rRNA gene sequence taxonomic identification using EzTaxon (6) confirmed that strain Bi-70 is most closely related to *Tersicoccus phoenicis* DSM 30849^T^ (98.8% identity). The genome sequences of *T. phoenicis* DSM 30849^T^ and *Tersicoccus* sp. Bi-70 consist of 132 and 19 scaffolds (total length, 3,210,299 bp and 3,479,893 bp; and *N*_50_, 108,378 bp and 556,029 bp, respectively) with median read coverages of 49.1 and 118.0×, respectively. The estimated genome sizes of both strains were smaller than those of sequenced strains belonging to the genus *Arthrobacter*, which is the closest relative of the genus *Tersicoccus* (e.g., 5.20 Mbp for *A. crystallopoietes* DSM 20117^T^ [accession no. GCA_900100805.1] and 4.95 Mbp for *A. globiformis* NBRC 12137^T^ [GCA_000238915.2]), and the G+C contents (DSM 30849^T^, 70.7%; Bi-70, 71.8%) were significantly higher than those of the *Arthrobacter* strains (DSM 20117^T^, 64.4%; NBRC 12137^T^, 66.2%).

For *Tersicoccus* sp. Bi-70, the PGAP identified 3,185 genes, including 3,128 protein-coding sequences (CDSs), 46 tRNA and three noncoding RNA genes, and 104 pseudogenes. The RAST annotation also identified a similar number of CDSs (3,204 CDSs). For *T. phoenicis* DSM 30849^T^, and the PGAP identified 3,088 genes, including 3,025 CDSs (2,991 CDSs by RAST), 46 tRNA and three noncoding RNA genes, and 152 pseudogenes.

The results of the RAST annotation showed that strains DSM 30849^T^ and Bi-70 possess only four and three ion acquisition and metabolism-related genes, respectively. The numbers of genes for these categories are much lower than those in the members of the genus *Arthrobacter* (26 in *A. crystallopoietes* DSM 20117^T^ and 22 in *A. globiformis* NBRC 12137^T^). However, despite the smaller genome size, the genus *Tersicoccus* has a nearly equivalent number of genes for other categories, e.g., 83 (DSM 30849^T^) and 91 (Bi-70) genes for the category of cell wall and capsule were identified in the *Tersicoccus* strains, while 104 (DSM 20117^T^) and 92 (NBRC 12137^T^) genes were identified in the *Arthrobacter* strains. The smaller genome sizes, the higher G+C contents, and the reduced numbers of ion acquisition and metabolism-related genes are the most characteristic features between members of the genera *Tersicoccus* and *Arthrobacter*. More detailed comparative analysis of the two *Tersicoccus* genomes will provide insights into the physiology and mechanisms of the adaptation to different environments.

### Accession number(s).

The whole-genome shotgun projects of *Tersicoccus phoenicis* DSM 30849^T^ and *Tersicoccus* sp. Bi-70 NBRC 112423 have been deposited in DDBJ/EMBL/GenBank under the accession numbers MRDE00000000 and MIEU00000000, respectively. The versions described in this paper are the first versions, MRDE01000000 and MIEU01000000.
